# Cystoma of Jaw

**Published:** 1891-08

**Authors:** Thos H. Moore

**Affiliations:** Philadelphia


### Cystoma of Jaw.

Mr. I., 46 years old, presented himself at my office, having a tumor involving the left half of the inferior maxilla. The growth, he says, began several months prior to his coming for treatment, and had attained considerable proportions. The cyst extended from the angle of the jaw, almost to the median line at the symphysis. The swelling was first noticed upon the lingual portion of the jaw, opposite the second inferior molar. It gradually increased in size, and then made its appearance upon the buccal surface and extended, anteriorly and posteriorly, involving nearly the whole left half of the jaw.

No reasonable doubt can be entertained but that the teeth are in most cases, the primary cause of such mischief. But the question of the precise manner in which the morbid conditions are developed is more difficult of solution. There is no disputing the fact that foreign bodies, such as the apices of teeth, broken off during extraction, or small spiculæ of bone, attracting a serous fluid around them, finally become encapsulated or encysted and gradually extend to great proportions.

Cystic tumors of the inferior maxilla are by no means of common occurrence, and present special features, such as slowness of growth, absence of severe pain, glandular involement, or constitutional impairment, except such as attends any tumor interfering with mastication or deglutition. The manner of discovering their true nature is by the aid of the exploring needle. In the present case I wish to say that the patient being possessed with a very fine set of natural teeth, none of them being devitalized, imagined they could be used for almost any purpose other than that for which they were intended.

He placed a hard shellbark between the two second molars of the left side of the jaw and cracked it. Immediately after, he experienced a slight pain in the lower tooth. This was evidently the cause of the formation of the tumor. The excessive pressure upon the cancellated bone injured the endostium, producing an effusion, and that is what formed the mucleus for an extended, but slow, inflammatory action, with the consequent results.

Obviously, there is but one sort of treatment for these tumors, and that is surgical. When they have not extended or reached to such proportions as to completely destroy the contour of the bone, I make a free incision to evacuate the contents. In the case being described this consisted of a dirty, bran-like substance, mingled with a glairy blood-stained fluid.

I made a further examination with the exploring needle, and discovered another but smaller cyst posterior to the one just opened, and having its connection by a small semis opening into the first one. I evacuated its contents also, and proceeded to wash out the cysts with tepid water. I then injected a stimulating solution of dilute tincture of iodine, and packed the cavity with gauze, which I removed, repeating the injection of the dilute iodine for a few days, until complete closure was accomplished. No internal medication was required in this case.

By Thos H. Moore, D.D.S., M.D.
Philadelphia.

